# Inhibition of caspase pathways limits CD4^+^ T cell loss and restores host anti-retroviral function in HIV-1 infected humanized mice with augmented lymphoid tissue

**DOI:** 10.1186/s12977-024-00641-2

**Published:** 2024-05-02

**Authors:** Alex J. Holloway, Tais B. Saito, Kubra F. Naqvi, Matthew B. Huante, Xiuzhen Fan, Joshua G. Lisinicchia, Benjamin B. Gelman, Janice J. Endsley, Mark A. Endsley

**Affiliations:** 1 Department of Microbiology and Immunology, University of Texas Medical Branch, 77555 Galveston, TX USA; 2 Department of Pathology, University of Texas Medical Branch, 77555 Galveston, TX USA; 3 Current at the Laboratory of Bacteriology, Rocky Mountain Laboratories, Division of Intramural Research, National Institute of Allergy and Infectious Diseases, National Institutes of Health, 59840 Hamilton, MT USA; 4 Present Address: Department of Internal Medicine, University of Texas Southwestern Medical Center, 75390 Dallas, TX USA; 5 Present Address: Department of Medicine, University of Toledo, 43614 Toledo, OH USA

**Keywords:** HIV-1, Lymphoid tissue, Caspase − 1, Pathogenesis

## Abstract

The study of HIV infection and pathogenicity in physical reservoirs requires a biologically relevant model. The human immune system (HIS) mouse is an established model of HIV infection, but defects in immune tissue reconstitution remain a challenge for examining pathology in tissues. We utilized exogenous injection of the human recombinant FMS-like tyrosine kinase 3 ligand (rFLT-3 L) into the hematopoietic stem cell (HSC) cord blood HIS mouse model to significantly expand the total area of lymph node (LN) and the number of circulating human T cells. The results enabled visualization and quantification of HIV infectivity, CD4 T cell depletion and other measures of pathogenesis in the secondary lymphoid tissues of the spleen and LN. Treatment with the Caspase-1/4 inhibitor VX-765 limited CD4^+^ T cell loss in the spleen and reduced viral load in both the spleen and axillary LN. In situ hybridization further demonstrated a decrease in viral RNA in both the spleen and LN. Transcriptomic analysis revealed that in vivo inhibition of caspase-1/4 led to an upregulation in host HIV restriction factors including SAMHD1 and APOBEC3A. These findings highlight the use of rFLT-3 L to augment human immune system characteristics in HIS mice to support investigations of HIV pathogenesis and test host directed therapies, though further refinements are needed to further augment LN architecture and cellular populations. The results further provide in vivo evidence of the potential to target inflammasome pathways as an avenue of host-directed therapy to limit immune dysfunction and virus replication in tissue compartments of HIV^+^ persons.

**Supplementary Information:**

The online version contains supplementary material available at 10.1186/s12977-024-00641-2.

## Introduction

In 2023, 39 million people were living with HIV worldwide [1]. During that year, 1.3 million people became newly infected and there were 630,000 HIV-related deaths [1]. Opportunistic co-infections such as tuberculosis exacerbate disease severity and lead to significantly worse outcomes in HIV^+^ patients [2–5]. Recent meta-analysis of people with HIV (PWH) has further revealed a greater risk for death due to COVID-19 [6].. Anti-retroviral therapies (ART) are highly effective and can control the disease, however, effective new prophylactics are still needed due to viral escape and anti-retroviral drug resistance. Biologically relevant in vivo models in which to test therapeutic candidates are restricted due to the host-species tropism of HIV [7]. The human immune system (HIS) mouse model reproduces many important endpoints of HIV disease but is challenged by suboptimal reconstitution of the murine spleen with human cells and a lack of well-formed LN [8, 9]. Secondary lymphoid organs, such as the spleen, LN, and draining lymphatic vessels, allow for the immune surveillance of the periphery and provide the structural framework for the physical interaction and communication of leukocytes. Early stage HIV infection is associated with an enlargement of LN that resolves following administration of ART, while long-term infection often results in fibrosis and significant impairment of LN function [10, 11]. Therefore, secondary lymphoid organs such as the spleen and LN are critically important for studying the pathology of HIV infection.

The most commonly used HIS mouse model is an irradiated and immune-deficient animal background in which human CD34^+^ cord blood stem cells are xenografted and allowed to reconstitute a human immune system. Human T cells, monocytes, macrophages, B cells and dendritic cells have been shown to develop in the blood and other tissues of this model [12–15]. HIS mouse models are well established to support HIV infection with resulting CD4^+^ T cell depletion and exacerbated disease in the setting of Mtb infection [16–21]. One shortcoming of this model, however, is that spleen and especially LN are poorly reconstituted [22–25].

We sought to optimize a HIS mouse model of HIV infection utilizing exogenous rFLT-3 L. FLT-3 L is a hematopoietic growth factor and is critical in normal hematopoiesis, growth, maintenance, and proliferation of all hematopoietic stem cells (HSC) cells. FLT-3 L is structurally homologous to stem cell factor (SCF) and has a strong synergistic effect with other cytokines on myeloid or lymphoid cell expansion. This cytokine is produced by all CD34^+^ cells, however the receptor (a member of the third class of receptor-like tyrosine kinases) is expressed on HSC and progenitor cells only [26–29]. The importance and functionality of FLT-3 L in hematopoiesis and the resulting reduced populations of dendritic and NK cells was demonstrated in a FLT-3 L knockout model [30]. Others have shown the effects of exogenous human FLT-3 L administration in mice and the resulting development and enhanced generation of human dendritic cell subsets in bone marrow and spleen [31, 32]. A transgenic mouse model constitutively expressing FLT-3 L further demonstrated this effect, producing enhanced B cell and myeloid cell populations as well as improved lymph node development [33]. In the current study, rFLT-3 L was employed to augment lymphoid tissue composition and size to establish a more robust HIV infection platform, and subsequently examine outcomes of host directed therapy targeting the inflammasome cascade in HIV infected HIS mice. The ability to use exogenous cytokine would provide a level of flexibility that is not available in a transgenic mouse model, allowing investigators to apply the benefits of FLT-3 L supplementation to animals of disparate backgrounds.

HIS mouse models with enhanced lymphoid tissue development are needed to facilitate investigation of HIV disease pathogenesis that are currently limited to in vitro models. Availability of an improved animal model for use in pre-clinical evaluation of drugs and therapeutics for people with HIV would represent an especially important advance. An important therapeutic target of interest for host directed approaches for HIV are the inflammasome pathways. Active caspase-1, a downstream component of the inflammasome, mediates the cleavage of IL-1β and IL-18 precursors into the active pro-inflammatory forms [34]. Caspase-1 is also responsible for the cleavage of gasdermin D which results in the programed form of cell death known as pyroptosis [35]. Additionally, the pyroptotic driven production of active IL-1β drives the cycle of HIV pathogenicity resulting in chronic inflammation [36–38]. It has been shown that > 95% of lymphoid CD4^+^ T cells die by caspase-1 mediated pyroptosis in an ex-vivo model of HIV infection in human tonsil and LN tissues, triggered by abortive viral infection [39].

These multiple roles of caspase-1 in both cell death and inflammation during an HIV infection offer promise for the efficacy of targeting caspase-1 or other inflammasome pathways. VX-765 is a potent inhibitor of enzymes caspase-1 and caspase-4 and reduces the production of IL-1β and IL-18 in models of inflammatory disease both in vitro and in vivo. It is a pro drug which after intake is metabolized into an active form [40], and has been shown to inhibit the release of IL-1β and IL-18 in peripheral blood mononuclear cells after stimulation with bacterial products without affecting secretion of other pro-inflammatory cytokines [41]. Oral administration of VX-765 has been shown to reduce the severity of disease and modulate cytokine secretion in a murine model of rheumatoid arthritis [42]. Importantly, VX-765 has been shown to prevent pyroptotic death in HIV infected human tonsillar and LN tissues infected with HIV [39]. Evidence for the in vivo efficacy of therapies that target inflammasome or other cell death pathways to reduce HIV pathogenesis is limited to date.

Herein we demonstrate that exogenous administration of rFLT-3 L enhances the development of LN and peripheral T cell populations in the CD34^+^ HIS mouse model. Further refinement of the model is still required to support optimal LN development, but this method holds promise for new avenues for discovery and testing of therapeutics. Additionally, we show that treatment of HIV infected mice with the caspase-1/4 inhibitor VX-765 reduces CD4^+^ T cell loss and viral replication in secondary lymphoid tissues. Interestingly, we also observed an increase in expression of host viral restriction factors upon caspase inhibition. These studies pave the way for further investigation into the therapeutic potential of caspase inhibitors in HIV infection.

## Results

### Effect of rFLT-3 L treatment on leukocytes in blood and tissue compartments of HIS mice

HIS mice were successfully generated following conditioning irradiation and engraftment of CD34 + human cord blood stem cells. To reduce potential for bias in interpretation of treatment effects, animals were assigned to treatment groups by matching peripheral blood reconstitution of human CD45^+^ and CD3^+^ leukocytes in individual animals as well as the group average planned for comparison. We examined the effect of rFLT-3 L administration on the size of lymphoid and myeloid cell compartments in the blood, LN and spleen of HIS mice.

**Fig. 1:**
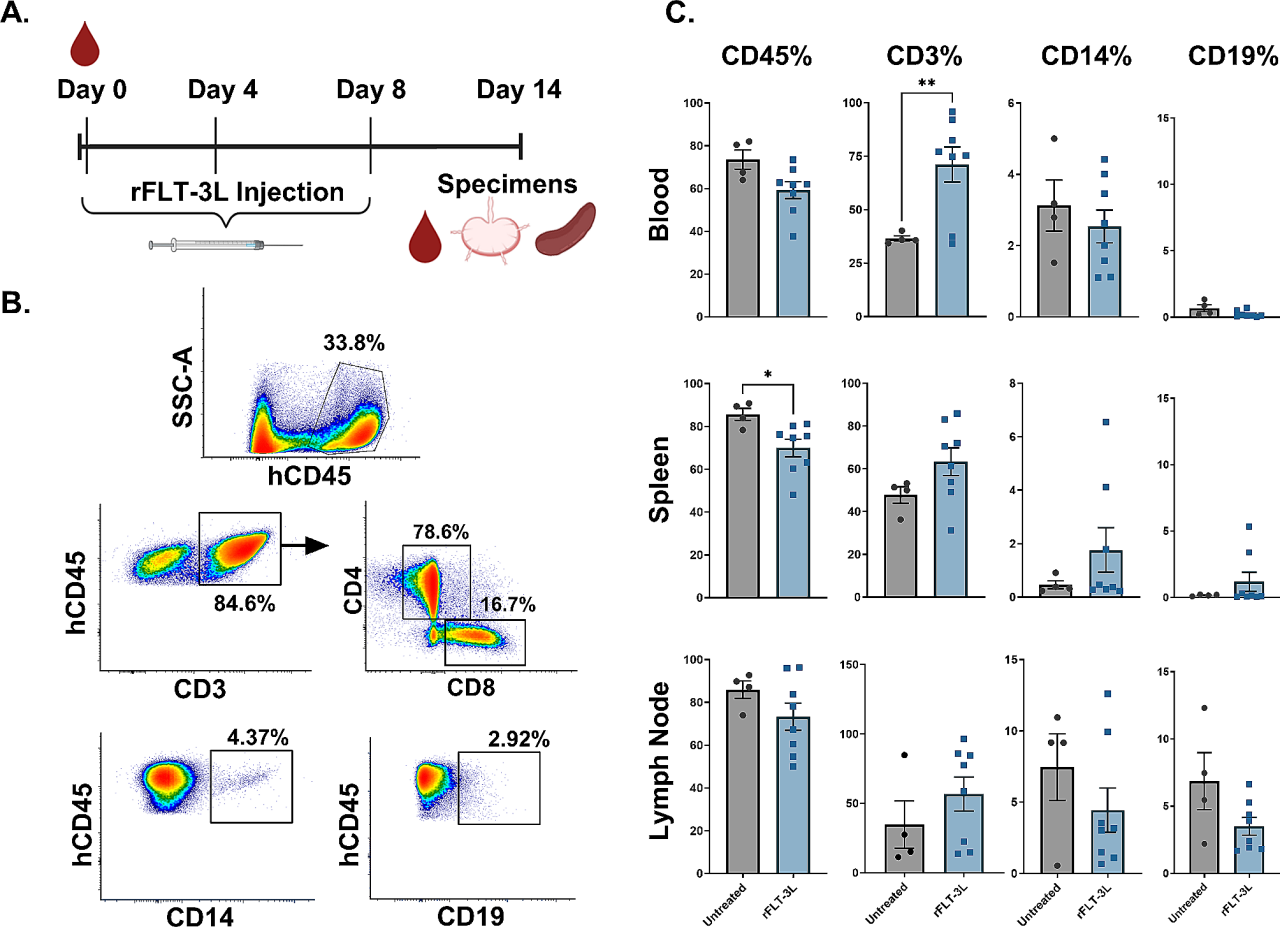
Effect of rFLT-3 L treatment on leukocytes in blood and tissue compartments of HIS mice. HIS mice were generated by engraftment of cord blood-derived stem cells into NSG mice at 3–4 weeks of age following conditioning irradiation of (125 cGy). Following reconstitution of human leukocyte populations, animals were distributed into groups (untreated *n* = 4, rFLT-3 L *n* = 8) and (**A**) rFLT-3 L was administered via subcutaneous injection (50ug in 250ul PBS, 0.2 mg/mL) once per day on days 0, 4 and 8. LN and spleen were harvested on day 14 for analysis via flow cytometry. Shown are (**B**) the gating strategy used to select human CD45^+^ leukocytes and further assess distribution of CD3^+^ T, CD14^+^ myeloid, and CD19^+^ B cell populations. (**C**) Summarized data (mean ± SEM) of the total percentages of human CD45^+^ cells within the leukocyte gate and the CD3^+^, CD14^+^ and CD19^+^ cells as a % of the human CD45^+^ population from the blood, spleen or axillary LN as observed at day 14 resulting from no treatment or administration of rFLT-3 L. Statistical comparisons were made using an unpaired two tailed Student’s T-test with Welch’s correction to determine significance of differences (*p < 0.05, **p < 0.01) due to treatment

Axillary LN were harvested and analyzed via flow cytometry and histology on day 14 following subcutaneous (s.c.) rFLT-3 L administration (50ug/mouse, *n* = 8 mice) on days 0, 4 and 8 as seen in the study design in Fig. 1A. Measurable LN were often not found in non-rFLT-3 L treated mice (*n* = 4), but tissue samples that were consistent with underdeveloped LNs were collected from LN sites (Figure S1) and processed for flow cytometric analysis. Right side axillary LN were harvested for flow cytometry, while left side axillary LN were used for histopathology and RNAScope to detect HIV transcription. Flow cytometric analysis of hCD45^+^ cells in the LN in the plot in Fig. 1B showed generation of distinct sub-populations of T cells (CD45^+^CD3^+^) including CD4^+^ and CD8^+^ subpopulations, monocytes/macrophages (CD45^+^CD14^+^), and B cells (CD45^+^CD19^+^) that develop in HIS mice (Fig. 1C). The overall % of leukocytes that express human CD45 declined moderately due to rFLT-3 L treatment, an effect that reached significance in the spleen. This effect may be due to additional effects of rFLT-3 L on proliferation or survival of murine cells, given that the % human CD45 cells is measured relative to the total leukocytes. Further analysis showed a significant increase in % CD3^+^ T cells in the blood in the rFLT-3 L group as compared to control, as well as a modest upregulation in the spleen and LN on day 14, though significance was not reached in tissues due to animal variance. In contrast to previous observations in a FLT-3 L transgenic mouse model, we observed no significant changes in the percentages of myeloid (CD14^+^) populations [43]. The overall numbers of human leukocytes including T cells or myeloid cells also remained unchanged following rFLT-3 L treatment (Figure S2). Similarly, populations of CD19^+^ B cells in the blood, spleen and LN were unchanged following rFLT-3 L treatment.

### Exogenous FLT-3 L treatment promotes development of lymph nodes that support HIV infection

**Fig. 2:**
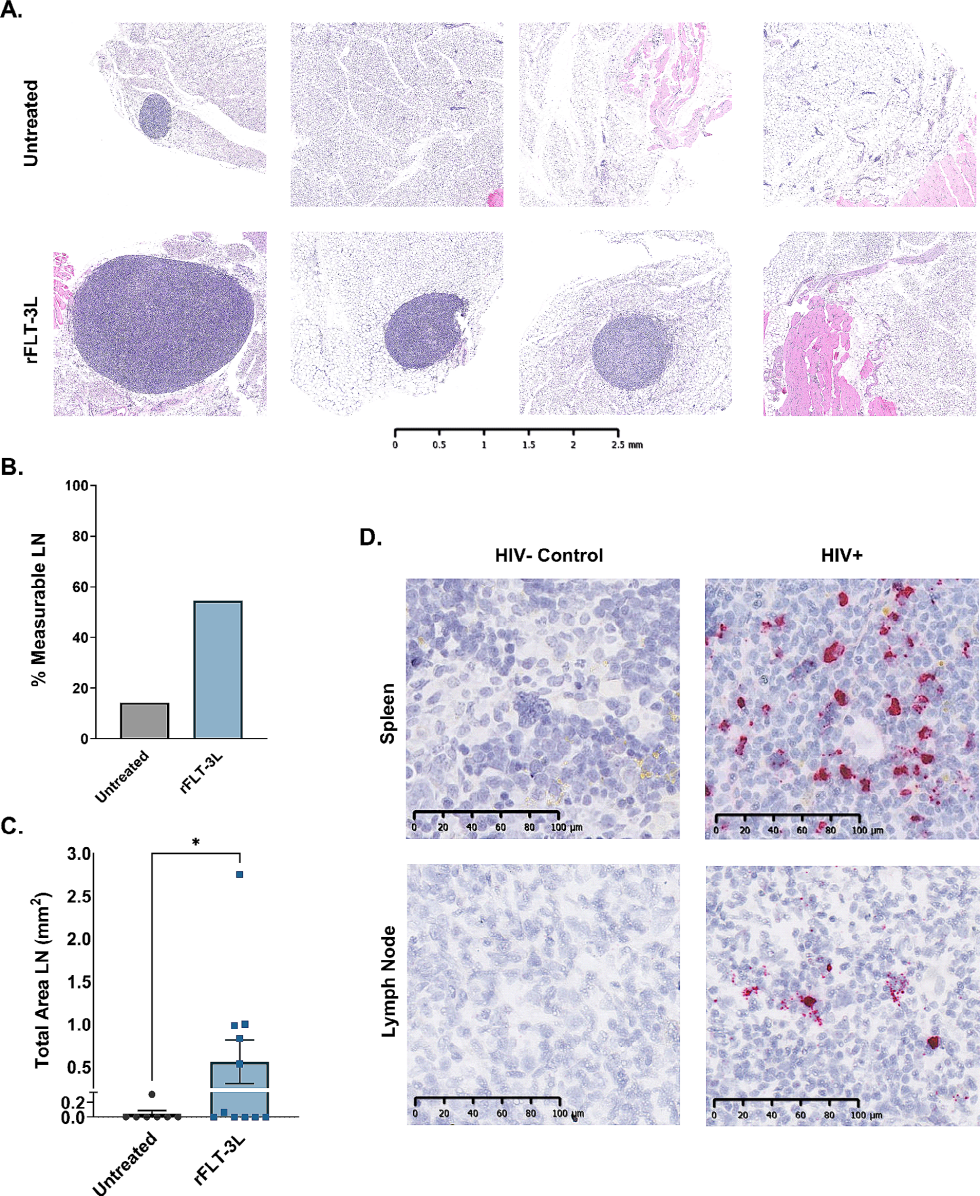
Exogenous FLT-3 L treatment promotes development of lymph nodes that support HIV infection. (**A**) H&E staining of representative axillary lymph node sections on day 14 post-administration of rFLT-3 L compared to non-treated animals. (**B**) Percentage of animals with measurable LN development following experimental rFLT-3 L treatment compared to controls. (**C**) Quantitation of the total area of LN development utilizing whole slide imaging and area analysis using QuPath software from the H&E image of untreated (*n* = 7) and rFLT-3 L treatment (*n* = 11) groups that were combined from two separate and identical studies. (**D**) Infection of rFLT-3 L-treated mice with HIV-1_ADA_ (10,000 ffu) demonstrates productive infection in the spleen and the LN as illustrated with representative tissue specimens following in situ hybridization to detect HIV *gag-pol* RNA. Significant differences due to treatment were determined using an unpaired two tailed Student’s T-test with Welch’s correction. Data is shown as the mean ± SEM. *p < 0.05

We further assessed the effects rFLT-3 L administration on the number and size of secondary lymphoid organs in untreated and rFLT-3 L-treated HIS mice which included tissue specimens from animals in replicate experiments. Examination of LN throughout the lymphatic system revealed an increase in the size of the axillary LN. Effects on development of mesenteric LN were also observed at lesser frequency compared to axillary LN, an effect likely due to proximity of axillary LN to the site of rFLT-3 L injection. Axillary LN were thus used for analysis for subsequent experiments and are subsequently designated as LN. HIS mice were treated as illustrated in Fig. 1A, and size and prevalence of LN was assessed at day 14 post-administration of rFLT-3 L. To compare sizes of LN, we sectioned each node through its largest dimension and analyzed the entire section of LN from each animal. Slides were scanned, and the area of each node was quantified planimetrically via QuPath software (Fig. 2). In non-treated mice, only 1 of 7 animals had measurable LN tissue in embedded tissue blocks (Fig. 2A, B). Presumptive leukocytes, based on flow cytometry outcomes, were present in lymphoid tissues of non-treated mice and were characterized by diffuse distribution of individual cells or small cellular aggregates as visualized in Figure S1A. LN were detected in 6/11 mice treated with rFLT-3 L, were more easily visualized (Fig. 2A), and were characterized by a more organized structure (Figure S1B) compared to tissues in non-treated control animals. The percentage of LN with measurable volume found in the rFLT-3 L group compared to non-treated mice was therefore increased as quantified in Fig. 2B. The organized tissue aggregates were also greater as determined by total area (mm [2]) as shown in Fig. 2C.

The same study design for augmentation of spleen and LN was used in subsequent studies of HIV, with HIV infection occurring on day 4. As shown with representative tissues from rFLT-3 L treated mice that were subsequently infected with HIV-1_ADA_ for 10 days (Fig. 2D) the LN were readily infected and produced detectable levels of HIV transcripts as measured using RNAScope and qPCR. As illustrated in Fig. 2D, however, HIV^+^ cells were markedly more abundant in spleen as compared to LN following assessment in tissue from infected and non-infected (control) animals. These improved LN, with further development, will provide broader opportunities to examine and evaluate antiretroviral and host directed therapies at a primary site of viral replication and T cell depletion.

### Caspase 1/4 inhibition with VX-765 limits CD4 ^+^ T cell loss in HIS mice infected with HIV

**Fig. 3:**
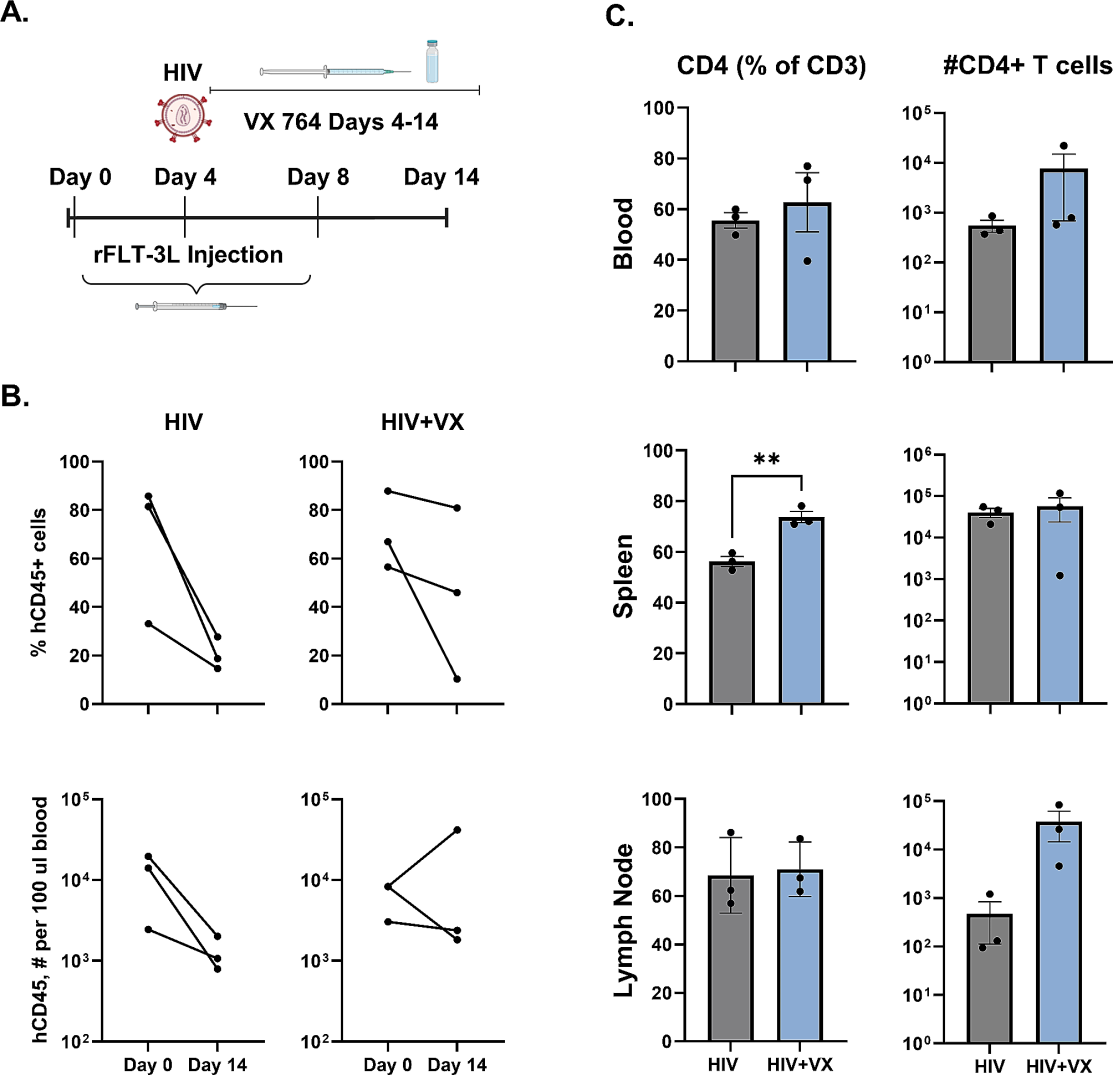
Caspase 1/4 inhibition with VX-765 limits CD4^+^T cell loss in HIS mice infected with HIV. HIS mice were selected for similar human immune system reconstitution and divided into two groups (*n* = 3/group). (**A**) Experimental design wherein both groups were administered rFLT-3 L (50ug in 250ul PBS, 0.2 mg/mL) on days 0, 4 and 8, and infected with HIV-1_ADA_ (10,000 ffu) on day 4. One group (HIV + VX) was treated with VX-765 via intraperitoneal injection on days 4–14 and one group (HIV control) was not treated. Blood, spleen, and LN were harvested on day 14 for analysis via flow cytometry. (**B**) Changes in human CD45^+^ leukocytes as a percent of total leukocytes and number of events in 100 ul of blood prior to HIV infection (d0) and at the end of study (d14) in HIV and HIV + VX groups. (**C**) Summarized flow cytometric analysis (mean ± SEM) demonstrating CD4^+^ cells as a percentage of human CD3^+^ T cells, and number of CD4^+^ cell events in the blood, spleen, and LN. Significant differences due to treatment were determined using an unpaired two-tailed Student’s T-test. **p < 0.01

To explore host directed therapy for an important mechanism of HIV pathogenesis, we treated HIS mice with rFLT-3 L and subsequently infected all animals with HIV as illustrated in Fig. 3A. As shown in Fig. 3B, a marked decrease in the percentage and number of peripheral blood CD45^+^ cells was detected by flow cytometry at 14 days post-infection with HIV compared to the day 0 pre-infection measurements of the same animal. Caspase 1/4 inhibitor VX-765 (100 mg/kg) treatment or no treatment was provided on days 4–14 as illustrated in Fig. 3A, followed by examination of blood, spleen, and LN leukocyte cell populations. Treatment with VX-765 partially alleviated the loss of human CD45^+^ leukocytes in the blood of HIV infected HIS mice from d0 to d14 when compared to non-treated animals. Flow cytometry analysis revealed that CD4^+^ T cells, as a percentage of T cells, were significantly greater in spleen of VX-765 treated mice as compared to non-treated controls. Similarly, the number of CD4^+^ T cells trended higher in LN of VX-765 treated mice although the CD4^+^ T cells as a percentage of CD3^+^ T cells was unchanged. No changes to CD4^+^ T cells were observed in peripheral blood (Fig. 3C). In a follow up study where CD4^+^ T cell loss due to HIV infection was less marked (Figure S3A), VX-765 treatment did not significantly affect the abundance of CD4 + T cells in general including as a % of the total CD3 + T cells (Figure S3B). These outcomes may reflect differences in HIV proliferation or host genetics of the stem cell source.

### HIV-1 replication is reduced by administration of VX-765 in an animal model of infection

**Fig. 4:**
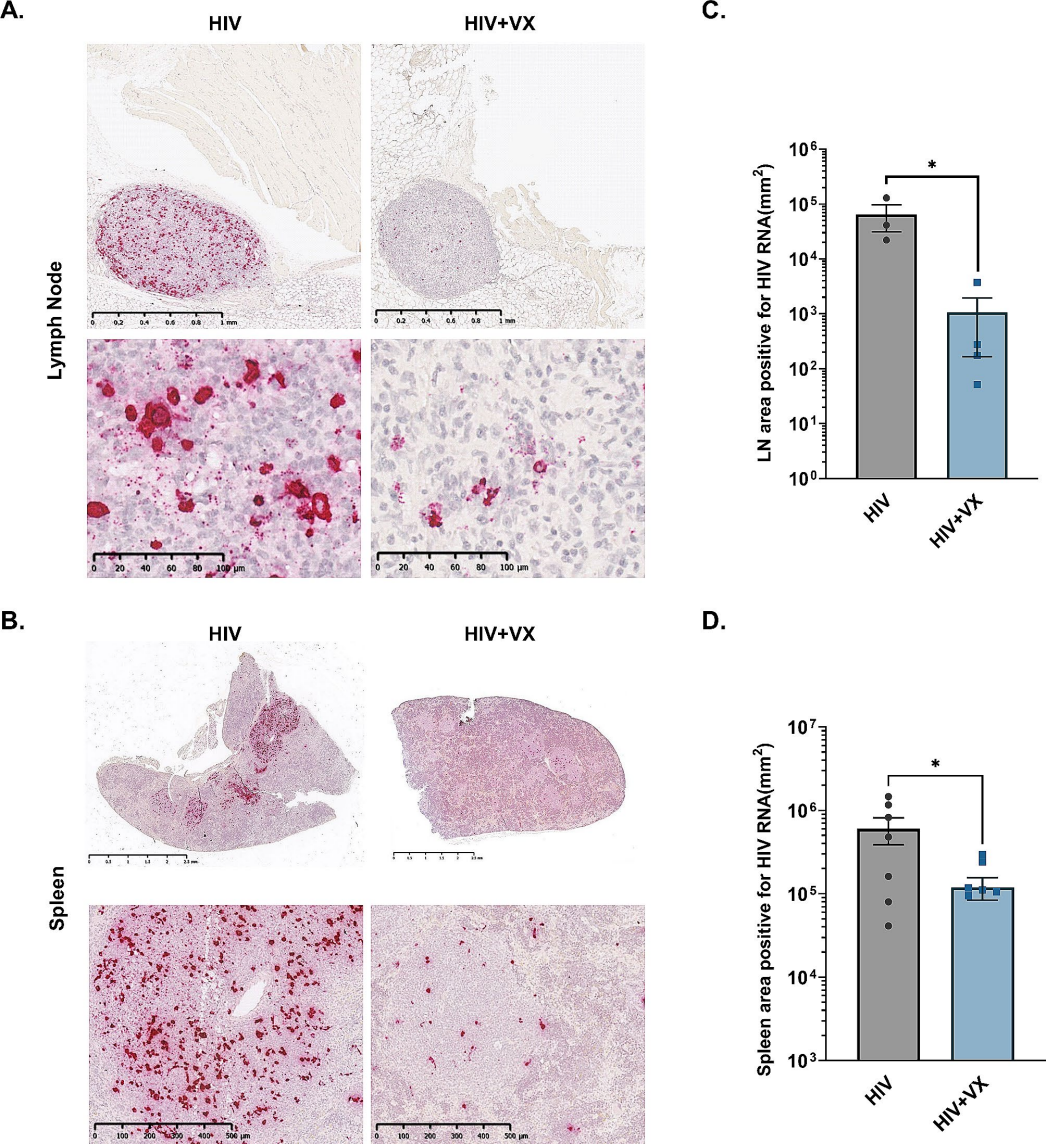
HIV-1 replication is reduced by administration of VX-765 in an animal model of infection. HIS mice were administered rFLT-3 L (50ug in 250ul PBS, 0.2 mg/mL) on days 0, 4 and 8, and infected with HIV-1_ADA_ (10,000 ffu) on day 4. One group (HIV + VX, *n* = 6) was treated with VX-765 via intraperitoneal injection on days 4–14 and one group (HIV control, *n* = 7) was not treated. Bone marrow, blood, spleen, and LN were harvested on day 14 for analysis via histopathology and RNAScope in situ hybridization with probes specific to HIV-1 *gag.* Representative imaging acquired from whole slide scanning of (**A**) axillary LN or (**B**) spleen, in prepared sections cut from the widest area of the tissue. Quantification of the resulting images was performed using Qupath software to quantify total area positive for viral RNA in (**C**) the animals where sufficiently developed axillary LN could be isolated (HIV *n* = 3, HIV + VX *n* = 4), and (**D**) in spleen of all treated animals (HIV *n* = 7, HIV + VX *n* = 6). Data is shown as endpoints from individual animals, in addition to summarized data presented as mean ± SEM. Significant differences due to treatment were determined using an unpaired two tailed Student’s T-test. *p < 0.05

In addition to assessment of CD4 + T cell changes, we further evaluated how inhibition of caspase 1/4 pathways would affect HIV replication. Mice were treated using the study design shown in Fig. 3A, and 1/2 of the spleen and a LN assayed for viral RNA by in situ hybridization. Caspase-1/4 inhibition had significant effects on the expression of HIV-1 RNA in tissues. Quantification of whole slide scans of in situ hybridization of HIV *gag* RNA revealed a significant decrease in viral RNA in both the LN (Fig. 4A, C) and spleen (Fig. 4B, D) of VX-765 treated mice. This was further supported by results of RT-PCR of total splenic RNA for HIV *gag* (Figure S4), which demonstrated a trend (*p* = 0.053) of reduced HIV *gag* RNA in spleens of VX-765 treated animals.

### Host viral restriction factors are increased by in vivo treatment with VX-765

**Fig. 5:**
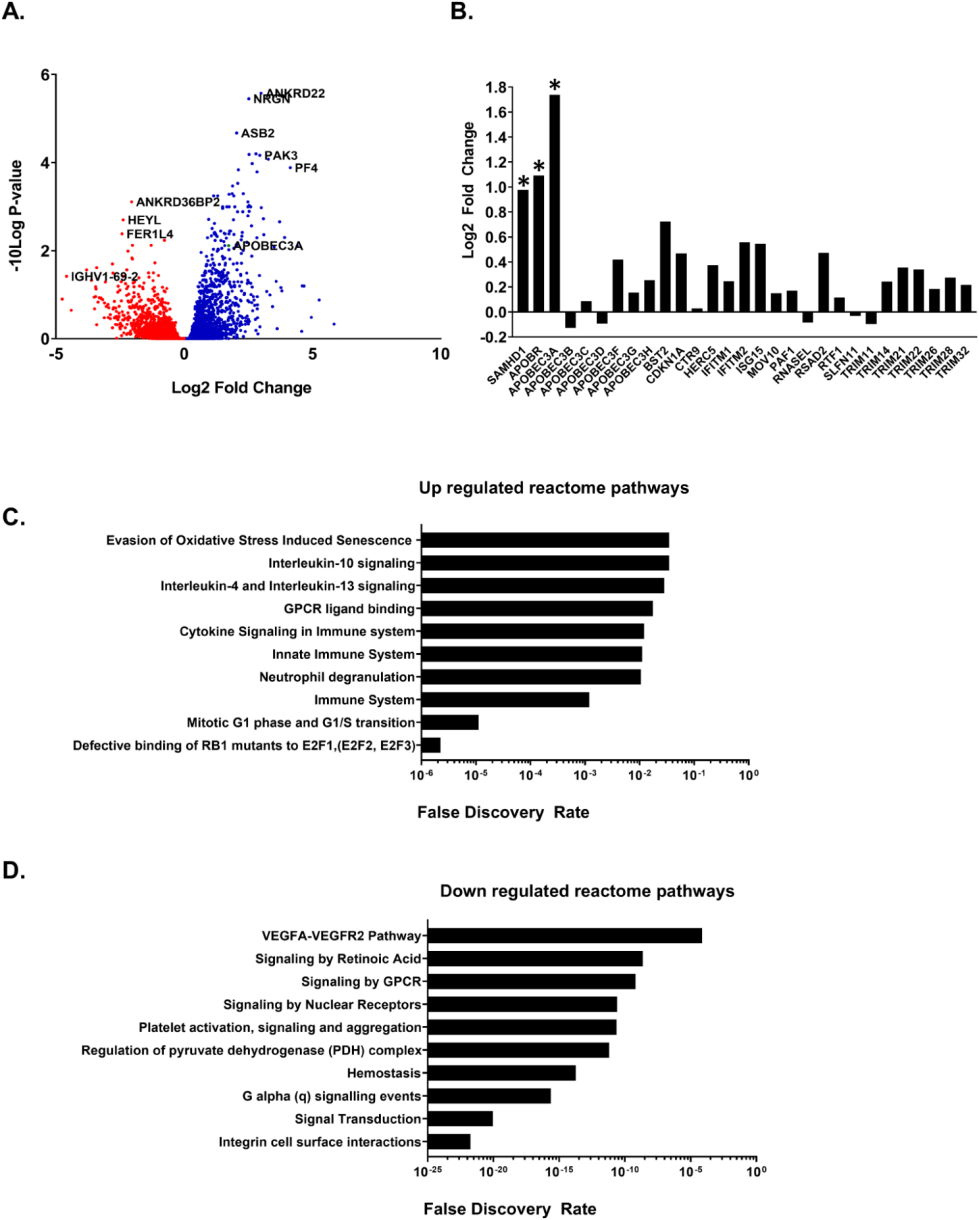
Host viral restriction factors are increased by in vivo treatment with VX-765. RNA was extracted from spleen of rFLT-3 L-treated mice that were infected with HIV (*n* = 3) or infected with HIV and treated with VX-765 (*n* = 3) following tissue collection on day 14 as described in Fig. 3A. (**A**) The differential transcriptome of HIV-infected mouse spleen, due to VX-765 treatment, was determined through expression analysis and visualized in a volcano plot with cut-offs at a p-value of < 0.05. (**B**) RNA-Seq analysis further elucidated several HIV restriction factors which were up or down regulated in the spleen mice in the HIV + VX-765 group compared to the HIV group. (**C**, **D**) Reactome Pathway Analysis of the differentially expressed genes was performed using String-db.org. The graphs represent up and down regulated pathways and biological processes predicted to result from VX-765 treatment of HIV-infected animals

To determine if other mechanisms might influence HIV RNA expression, we compared the transcriptome of spleen from VX-765 treated, HIV-infected animals to those with HIV alone (Fig. 5A). Several viral restriction factors were upregulated in the HIV^+^ VX-765 treated group compared to HIV-infected controls (Fig. 5B). Important anti-retroviral factors SAMHD1and APOBEC3A were all significantly upregulated (between ∼ 2 and ∼ 4 times) in the presence of VX-765. Several other antiviral restriction factors were also upregulated (BST2, IFITM2, ISG15) but did not reach significance (Fig. 5B). Reactome pathway analysis of splenic RNA-Seq results identified multiple upregulated reactome pathways effected by caspase-1/4 blockade (Fig. 5C, D), including those involved in general and innate immune system activation, neutrophil degranulation, cytokine signaling, G-protein coupled receptor ligand binding, and evasion of senescence induced by oxidative stress (Fig. 5C). Additionally, several pathways were identified as downregulated including, integrin cell surface interactions, signal transduction, signaling by retinoic acid and nuclear receptors, regulation of pyruvate dehydrogenase complex, and wound healing pathways including hemostasis and platelet activation (Fig. 5D).

### Network analysis identifies immune pathway alterations in HIV-infected tissue due to VX-765 treatment

**Fig. 6:**
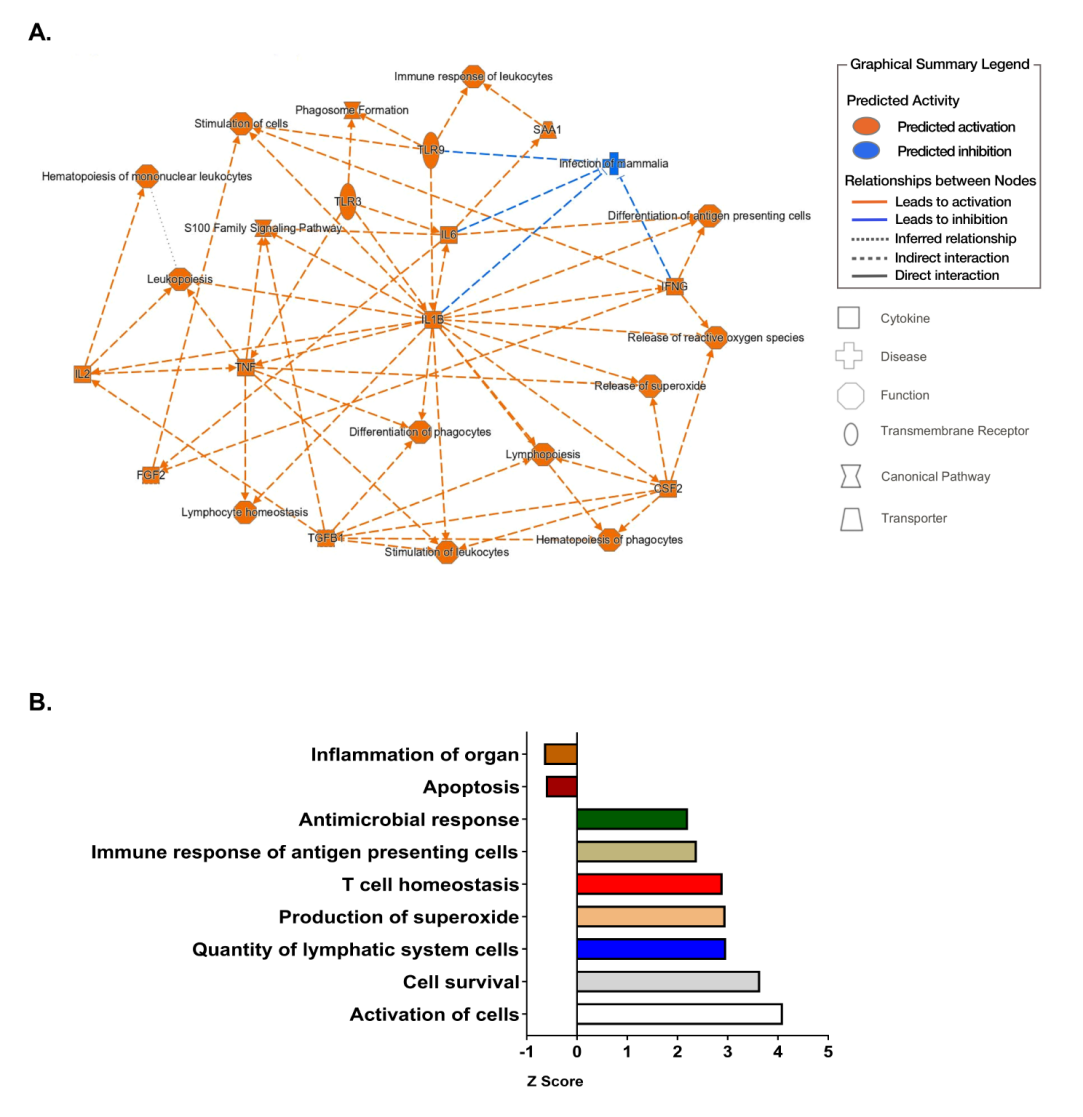
Network analysis identifies immune pathway alterations in HIV-infected tissue due to VX-765 treatment. Interconnected pathways that are disrupted due to in vivo inhibition of caspase 1/4 were determined in HIV-infected HIS mice that were non-treated (*n* = 3) or treated with VX-765 (*n* = 3) as described in Fig. 3A. Significantly perturbed pathways were identified through Ingenuity Pathway Analysis of RNA-Seq data from spleen using *p* ≤ 0.001 and Z score ≥ + 2 for activation or *p* ≤ 0.001 and Z score ≤ -2 for suppression

Ingenuity Pathway Analysis further revealed that VX-765 treatment disrupted a number of interconnected pathways related to immune outcomes, especially pro-inflammatory cytokine (IL-1β, IL-6 and TNF) activation (Fig. 6A). Downregulated pathways were associated with organ inflammation and apoptosis (Fig. 6B). Upregulated pathways were associated with antimicrobial immunity, and this was supported by increased expression of genes involved in neutrophil recruitment and activation (CXCR1, FPR1, FPR2, S100A8/9, and MPO), and antigen presenting cell responses. Other upregulated pathways involved cell survival and activation (Fig. 6B), including genes such as BCL6, KDR (VEGFR), SMO (Smoothened), and WNT5A. RNA-Seq data for individual genes has been uploaded into NCBI’s Gene Expression Omnibus repository, as described below.

## Discussion

The study of the complex interactions of HIV pathogenesis in an intact in vivo model is critical when attempting to elucidate disease mechanisms, as well as measuring the efficacy of potential therapeutics. The exclusively human tropism, rapid mutation rate and the ability of HIV to remain latent within the genome are especially difficult obstacles in efforts to find a cure or prophylactic. Tools allowing us to study HIV pathogenesis, such as non-human primate and humanized mouse models, have rapidly advanced our understanding of the roles of various immune cells and their physical interactions when examining specific tissues as well as the organism as a whole [20, 44–47]. Our present work highlights how optimizing lymphoid tissue compartments of the HIS mouse model with exogenous rFLT-3 L can improve its utility for the study of HIV pathogenesis and reservoirs in various tissue compartments, while showcasing the immunomodulatory protection offered by host-directed therapy with an inflammasome pathway modulator.

Lymph nodes provide a structural framework for the interaction between B and T lymphocytes, as well as other antigen presenting cells, which is critical to intercellular communication, immune surveillance and adequate interaction of cells and antigens necessary to generate a proper response to infection [48, 49]. This properly functioning arrangement is dependent upon the tissue structure, cell density and inflammatory state, all of which are critical variables in HIV pathogenesis. It is well established that HIV infection disrupts the architecture of the lymph node. While early HIV infection may involve lymphadenopathy, late-stage infection is often accompanied by smaller, fibrotic lymph nodes which are lacking lymphocyte populations and characterized by impaired fluid flow [11, 50–52]. HIS mouse lymph nodes are characteristically small, indistinct and are typically difficult to discern from surrounding adipose and connective tissue, making gross pathology analysis less than ideal in terms of biological relevance to human disease [53]. We observed an increase in the abundance and size of the lymph nodes found in animals with the administration of rFLT-3 L, while flow cytometry analysis revealed an increase in human CD3^+^ T cells. With this augmentation, we can begin to more closely recreate the well-developed and densely cellular human lymph node critical for pathology analysis during HIV infection.

As a demonstration of the model utility, we observed significant HIV viral replication in the LN and spleen on day 14 post-infection that corresponded with a decrease in CD4^+^ T cells in the peripheral blood. Further refinements to the model are still necessary, however, as fully reconstituted LN with organized germinal centers and other features have yet to be achieved. Optimization of this model could involve incorporating mice of various backgrounds such as the Balb/c *Rag2*^−/−^*Il2rg*^−/−^*Flt3*^−/−^. As there is cross species reactivity to rFLT3L, murine cells will be utilizing the exogenously delivered protein which is a limitation of our model. *Flt3*^−/−^ strains have been used as in the previously mentioned work by Li, Y. et al., in which their model showed selective enhanced development of human dendritic cells in response to rFLT-3 L administration. As a result of the *Flt3*^−/−^ and the resulting loss of hosts tyrosine receptor kinase, the exogenously administered rFLT-3 L is not in competition to for murine myelopoiesis which resulted in improved dendritic cell homeostasis and an increased number of human NK and T cells [31]. Outside of the mouse model, the work of Coats, PT et al. showed increases in dendritic precursor cell populations in the inguinal lymph nodes after rFLT-3 L administration in the rhesus macaque NHP model [54]. The work of Kremer IB. et al. also showed increases of dendritic cells in lymph nodes of BALB/c mice [55] which highlights the compositional changes of the secondary lymphoid tissues as a result of rFLT-3 ligand administration.

Pyroptosis, which is an inflammasome-initiated means of cell death mediated by caspase-1 activation, is one of the major mechanistic processes responsible for the depletion of CD4^+^ T cells during HIV infection. While antiretroviral therapies function to suppress viral load, they do not mediate inflammation and cell death by direct means. Also, due to the high mutation rate of the virus, resistance occurs in all current ARV [56, 57]. Therefore, therapeutics which function in a host mediated means as opposed to interference with viral processes offer distinct advantages. As VX-765 inhibits a host protein, it should offer broad protection against multiple strains and mutations. While VX-765 proved ineffective in a clinical trial for relief of epilepsy, it was demonstrated to be safe to use in humans [58]. Our work provides in vivo support for the findings of Doitsh, et al. [39]., which showed that inhibition of caspase-1 in HIV infected human tonsil explants limits CD4^+^ T cell loss. Additionally, a recent publication by Amand et al., demonstrated the role of VX-765 in abrogating T cell depletion in the NSG mouse model of HIV infection. Their observation that T cell depletion was decreased in the blood and bone marrow are in agreement with our findings, though they did not observe the restoration of T cells in the spleen that we have shown here. These outcomes may be due to experimental differences including the strain (JRCSF vs. ADA) and infectious dose of HIV used and both the dosage and duration of treatment with VX-765 [59]. The varying effects of VX-765 treatment on HIV-mediated depletion of CD45^+^ and CD4^+^ cells that we observed in different animals, within and between studies (Fig. 3 and Figure S3), may also suggest the need to optimize VX-765 for improved in vivo efficacy or explore other compounds to target inflammasome pathways. Importantly, our work expands the understanding of host directed therapy targeting the inflammasome in the LN compartment and demonstrates antiviral effects of treatment at this important site for HIV pathogenesis.

Our exploration of the differential transcriptome further provides the first insight into the mechanisms whereby VX-765 and other caspase inhibitors may promote antiviral immunity. Analysis of RNA-Seq data showed a decrease in cellular pathways associated with cell death (Fig. 6), and no corresponding increase in cell cycle proteins, arguing that changes in T cell numbers were due to a decrease in cell death rather than an increase in proliferation. As shown in Figure S3, preservation of CD4^+^ T cell counts were not observed in animals with an apparently milder course of HIV infection where CD4 + T cell loss was less marked. Further, VX-765 treatment resulted in a significant decrease in viral replication, likely due to a decrease in the release of bioactive IL-1β following ICE cleavage. An important contribution of our work is an observed up-regulation of several host viral restriction factors in response to caspase-1/4 inhibition. Reactome Pathway Analysis of RNA-Seq data identified innate and adaptive immune responses, as well as several molecules (e.g. SAMHD1, APOBEC3A) with roles as HIV host restriction factors, which were upregulated in the HIV + VX-765 treatment group compared to HIV alone. These HIV host restriction factors interfere with the various multistep processes of viral replication. SAMHD1 and APOBC3A interfere with reverse transcription prior to integration [60]. As these host restriction factors compose the first line of defense against viral replication, the implications of these findings could be significant [61–63]. We should note that of the potential list of HIV host restriction factors investigated in this experiment, the factors listed are likely only a small subset of critical factors in HIV infection as many more have yet to be identified and their implications explored. We have yet to elucidate the cellular mechanisms associated with these newly identified changes, but they could be due to the lack of inflammatory interference with type-1 interferon signaling [64].

As HIV mutations continue to be one of the primary obstacles in the development of a vaccine or treatment, elucidating the additional regulatory schemes of these host restriction factors could offer the insight needed to more broadly understand the initial impacts host cells play during the early stages of infection and viral replication. While there were no changes in IL-1β expression detected in RNA-Seq analysis, there were significant alterations to pathways related to IL-1β signaling. This is unsurprising, as caspase-1 inhibition prevents cleavage of pro-IL-1β into its active form and not upstream events that regulate transcription of IL-1β. Our observations demonstrate that in vivo inhibition of caspase 1/4 led to increased activation of genes in pro-inflammatory pathways (e.g. IL-1β, IL-6, TNF) that are regulated through TLR and NFkB signaling networks. Interestingly, these are pathways associated with signal 1 in the inflammasome cascade that promote IL-1β transcription. Activated caspase 1 (target for VX-765) is the end product of inflammasome complex assembly (signal 2) which cleaves pro-IL-1β to active IL-1β. These results may suggest that inhibition of caspase 1/4 may drive greater pro-inflammatory cytokine pathways as part of a feedback process. Given the crosstalk among IL-1β and Type I IFN pathways, the observed increase in host restriction factors following VX-765 treatment may reflect a stronger type I IFN response as a therapeutic benefit of limiting bioactive IL-1β [64]. Importantly, these changes in pro-inflammatory signatures due to caspase 1/4 inhibition were associated with diminished cell death pathway activation, preservation of CD4 + T cells, and reduced viral replication in HIS mice infected with HIV.

## Conclusions

**Fig. 7:**
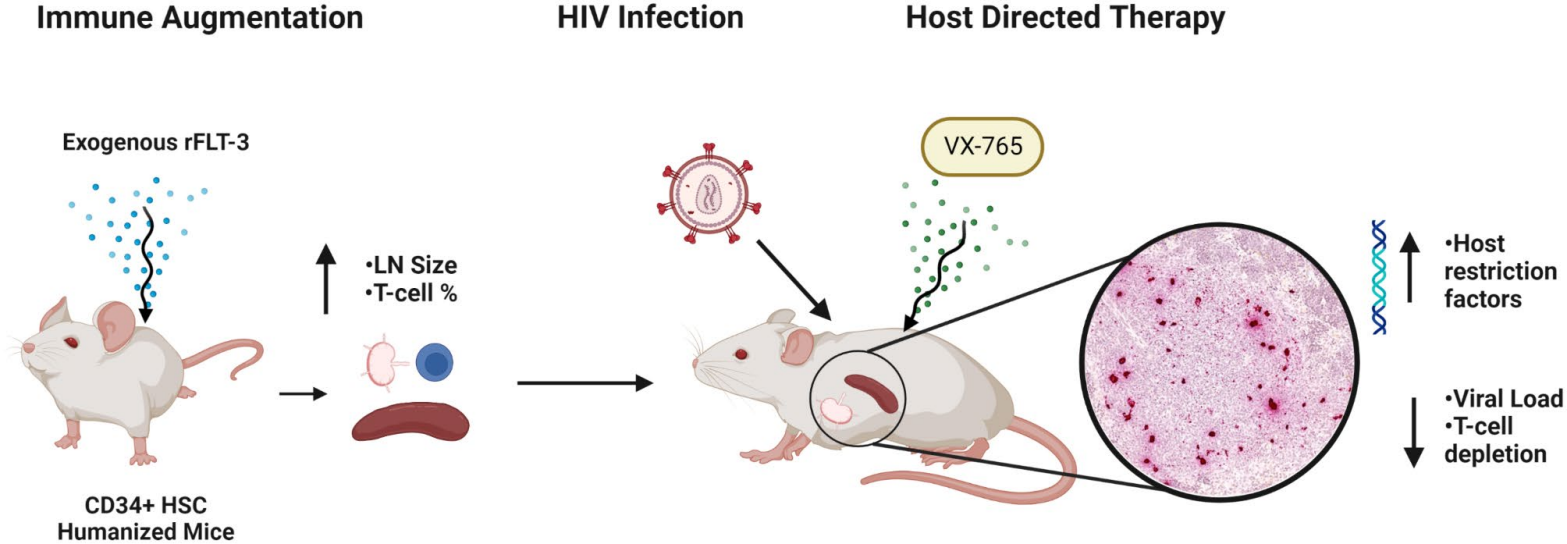
Graphical summary. Enhancements to the HIS mouse model that augment secondary lymphoid organs expand opportunities for experimentation to understand pathogenesis and test interventions in a small animal model of HIV-infection. Treatment with exogenous human rFLT-3 L promoted the development of LN and increased the relative abundance of human T cells in spleen of HIS mice. This advance permits investigations of HIV-mediated immune outcomes and tissue-level pathogenesis in highly relevant secondary lymphoid compartments. Targeting the inflammasome cascade in rFLT-3 L-treated and HIV-infected HIS mice through in vivo treatment with the caspase 1/4 inhibitor VX-765 improved disease outcomes associated with greater immune activation and reduced viral burden. HIS models require further refinement to generate lymphoid tissues which more closely recapitulate human tissues, but represent an important tool for discovery and translation for development of HIV interventions

Our work demonstrates that use of HIS mice with augmented secondary lymphoid tissue is an important advance for HIV discovery and translation research. Several refinements are still needed to achieve fully developed and normally distributed lymph nodes when using exogenously administered rFLT-3 L. Enhancements to the HIS mouse model through exogenous or transgenic rFLT-3 L provision nonetheless allow for mechanistic inquiry and testing of interventions in the context of HIV-infected secondary lymphoid tissues in a small animal model (Fig. 7). Exploration of host directed therapies targeting inflammasome pathways for use as an adjunctive to antiretrovirals or treatment of inflammatory conditions in PWH is further warranted.

## Methods

### Generation of humanized mice

Human immune system mice were generated by engraftment of cord blood-derived stem cells into immune deficient and irradiated mice. NOD.Cg-Prkdc^SCID^Il2rg^tm1Wjl^/SzJ (or NSG) mice (Jackson Laboratory) at 3–4 weeks of age received a sublethal radiation dose of 125 cGY exposure in a RS 2000 small animal irradiator. Mixed donor human cord blood CD34^+^ stem cells (Stem Cell Technologies) were rapidly thawed, washed with PBS + 5% FBS solution and resuspended in a sterile Diprotin A (R&D Systems) solution prior to injection. Following irradiation, mice were injected i.v. with 1 × 10^5^ CD34^+^ cells per mouse. Prior to human immune system reconstitution, mice were housed in sterile bedding, received gamma-irradiated feed, and acidified (HCL) drinking water to reduce potential for environmental opportunistic infections. Reconstitution of human leukocyte populations was evaluated in peripheral blood by flow cytometry as we have previously described [21] using cell surface markers specific to human CD45, CD3, CD4, CD8 and CD14.

### Immune modulatory treatments

Recombinant human FLT-3 ligand was purchased from Peprotech (Cranbury, NJ) and resuspended in sterile PBS at a concentration of 0.2 mg/ml. rFLT-3 L was administered once per day via subcutaneous injection of 50ug in 250ul PBS, divided into two injections in the upper and lower dorsal surface. Administration of rFLT-3 L occurred on days 0, 4 and 8, and effects evaluated at 14 days. The caspase-1 inhibitor VX-765 was purchased from MedChemExpress (Monmouth Junction, NJ). VX-765 was initially diluted into DMSO and subsequently diluted with H_2_0 containing 10%Tween and 10% DMSO for injection at a concentration of 100 mg/kg in 100 µl. Animals were treated once daily with VX-765 via intraperitoneal administration from days 4 to 14.

### HIV infection

The HIV-1_ADA_ was acquired from the NIH AIDS Repository and propagated in human peripheral blood mononuclear cells (PBMC), as described [65]. The PBMC were isolated from fresh buffy coats obtained from the Gulf Coast Regional Blood Center (Houston, Texas) and stimulated with PHAp (1ug/ml) and IL2 (2.5ng/ml) for 3 days. Following three days of activation, PBMC were infected with HIV in low volume media conditions for 1 h followed by incubation in full media for another 15 h. The media was subsequently replaced to remove non-infectious virus and the cells were incubated for 7 days with media changes occurring on days 2 and 4. On day 7, the supernatant was collected and centrifuged at 350 g x 10 min and quantified for TCID_50_ by the Spearman-Karber method and focus-forming units (ffu) by fluorescence measurement in GHOST cells [66, 67]. Stocks of the supernatant containing virus were stored at -80 °C. Stocks of HIV-1_ADA_ were diluted into sterile PBS to a final concentration of 100,000 IU/100uL and was administered via tail vein injection in 100uL volume.

### Histology

On day 14 post-infection, animals were humanely euthanized, and terminal blood collection was then performed via cardiac puncture for flow cytometry analysis. A portion of the spleen and one axillary lymph node were harvested and immediately placed into 10% neutral buffered formalin. After 24 h, samples were placed in fresh 10% neutral buffered formalin and samples were transferred to the UTMB Histopathology Core for paraffin embedding, sectioning (5 μm) and performance of hematoxylin and eosin staining. Additional tissue sections were used for performance of RNAScope imaging and analysis.

### Isolation of blood and cells from tissues

At necropsy, a portion of the spleen, and 1 of the 2 axillary lymph nodes (when available) was harvested and placed into transport medium (RPMI) for use in flow cytometric analyses. While not all animals developed measurable LN structures, sites where LN would be expected did contain lymphoid tissues with diffuse or small aggregate of cells (Figure S1A) that were harvested and analyzed. Blood was collected via retro-orbital sinus or cardiac puncture (day 0 and day 14 respectively) into ethylene-diamine-tetra-acetic acid (EDTA) hematology tubes. The spleen and lymph nodes were manually homogenized by pressing tissues through a 70 μm cell strainer. Disrupted cells and media used to rinse the cell strainer of residual cells were passed into a 50mL conical tube. The cells were subsequently strained again through a 40 μm strainer and washed in cPRMI prior to flow cytometric analysis.

### Multi-parameter flow cytometry

The cellular phenotype of cells from blood and tissue (lung and spleen) was determined using flow cytometry. Non-specific binding of detection reagents was controlled by 5 min incubation with an Fc receptor blocking reagent (FC block, BD Bioscience 553,142) prior to addition of detection antibodies. An optimized flow cytometry panel for detection of mouse CD45 (Biolegend 553,081) and human surface markers: CD45 (Biolegend 339,192), CD3 (Biolegend 563,546); CD4 (Biolegend 558,116); CD8 (Biolegend 341,051); CD14 (Biolegend 563,698); and CD19 (Biolegend 302,225) was prepared in brilliant stain buffer (BD 566,349). This staining panel was added to the cells and samples were incubated at room temperature for 30 min in the dark. After the staining step, red blood cells (RBCs) in single-cell suspension of spleen and LN were lysed by 5 min incubation with a commercial lysis buffer (Sigma 11,714,389,001). The FACS lyse buffer (BD 349,202) was used to lyse RBCs in the whole blood samples) by using a 10–15 min lysis step. Following RBC lysis, samples were centrifuged at 500 x g for 5 min to collect the white blood cell pellet and rinsed in 2mL of FACS buffer (Ca-Mg- free PBS, 5% BSA, 0.1% sodium azide) and centrifuge at 330xg for 5 min. The cells were then resuspended into 200µL of 2% UltraPure Formaldehyde (PolySciences Inc. cat# 18814-20) in PBS to inactivate HIV and placed at 4 °C protected from light until acquisition on the flow cytometer. Samples were acquired using a BD LSR II (Fortessa) flow cytometer (BD Biosciences) in the UTMB Flow Cytometry and Cell Sorting Core Facility, and data was analyzed using FCS Express6 (DeNovo).

### RNAScope

Spleen and axillary lymph node sections were used for analysis of viral RNA via RNAScope (ACD Bio) using in situ hybridization with probes specific to the HIV-1 *gag* gene in accordance with manufacturer’s instructions. Briefly, slides were deparaffinized with xylene and rehydrated a series of ethanol and PBS submersions. Heat mediated antigen retrieval was performed in target retrieval buffer (ACD Bio) for 10 min at 95 °C in an EZ-Retriever microwave (BioGenex). Slides were rinsed in 100% ethanol and dried overnight. Amplification buffers 1–6 (ACD Bio) were incubated at the time, temperature and duration according to manufacturer’s instructions. The chromogenic substrate was added to the tissues for 10 min at r/t following hematoxylin staining. Whole slide imaging was performed using the Hamatsu Nano Zoomer 2.0RS imaging system. Image quantification was performed using Qupath software to quantify total area positive for viral RNA.

### Quantification of HIV RNA

Total RNA was collected from pre-weighed spleen samples using a Zymo RNA extraction kit, and cDNA was produced using a Bio-Rad iScript cDNA Synthesis kit. HIV transcripts were assayed by SYBR Green RT-PCR using primers spanning the *gag-pol* junction [68] in the HIV genome and normalized to tissue weight in grams.

### RNA sequencing

RNA was extracted from spleen isolated on day 14 using the Aurum™ Total RNA Mini Kit with DNAse-1, as previously described [69]. RNA-Seq was performed on an Illumina NextSeq550 using the High-Output flow cell, single-end 75 base read format. A total of 465 million reads were obtained, and ranged from ∼ 40–60 million per sample. Proceeding library construction and mapping to the human reference genome, genes were sorted based on adjusted (adj.) p value and log2 fold change. Statistically significant (adj. p value < 0.05) upregulated or downregulated genes were selected for individual identification in volcano plots and for the listing of HIV host restriction factors (GraphPad Prism), in Reactome Pathway Analysis using STRING database (ELIXIR), and interconnected pathways alterations using Ingenuity Pathway Analysis (QIAGEN Digital Insights).

### Statistical analysis

All data were analyzed using GraphPad Prism version 10 and presented as mean (± SEM). Significant differences were determined using a one-way ANOVA followed by an appropriate post hoc test (e.g., Tukey’s) for differences due to treatment, or between treatment groups, as indicated in each figure legend. Statistical analysis of data sets containing only two experimental groups was conducted using a two-tailed unpaired T test. P-values of < 0.05 were considered significant.

### Electronic supplementary material

Below is the link to the electronic supplementary material.

Supplementary Material 1

## Data Availability

The dataset supporting the conclusions of this article is available in NCBI’s Gene Expression Omnibus (Edgar et al., 2002) repository, GEO Series accession number GSE213865.
