# Supplementary material for: Inhibition of caspase pathways limits CD4+ T cell loss and restores host anti-retroviral function in HIV-1 infected humanized mice with augmented lymphoid tissue
Source: Retrovirology. 2024 May 2;21:8. doi: 10.1186/s12977-024-00641-2 (PMC11064318; doi:10.1186/s12977-024-00641-2)
Supplement: Supplementary file 1 — Supplementary Material 1 [file 12977_2024_641_MOESM1_ESM.docx]

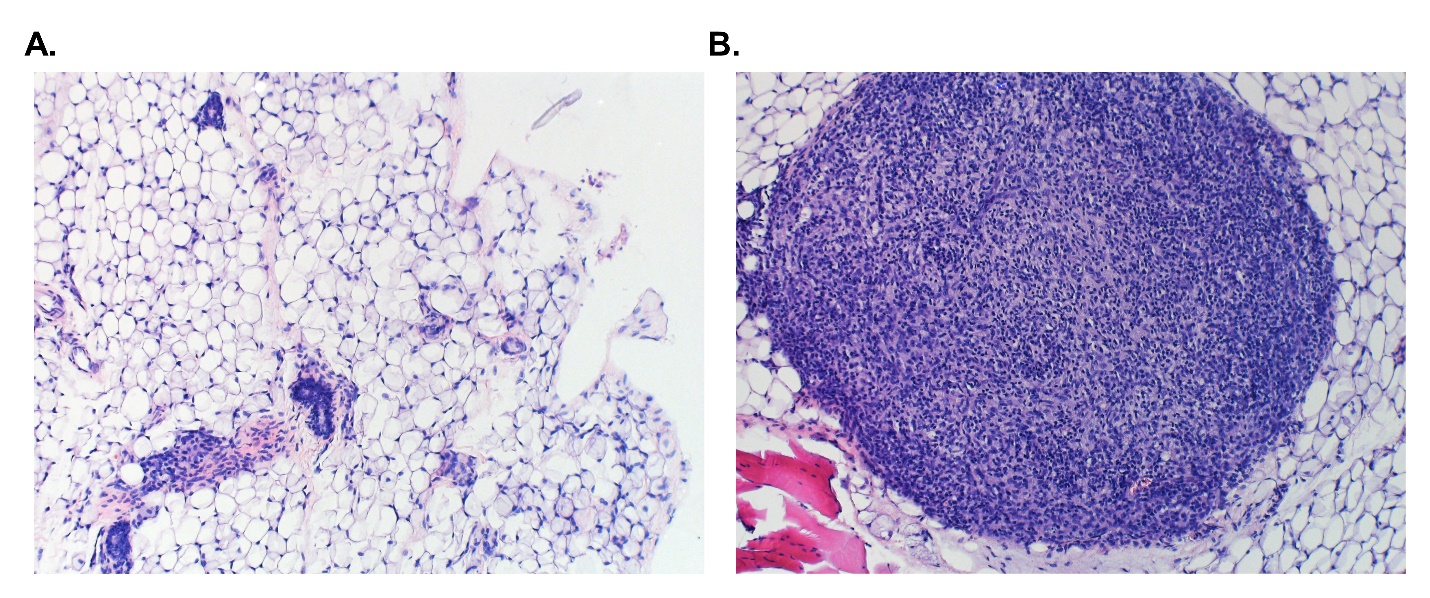


**Figure S1  Lymph tissue development in untreated or rFTL-3L-treated HIS mice.** H&E visualization of representative axillary lymphoid tissues collected on day 14 post-administration of rFLT-3L in non-treated (left) and treated mice (right). Tissues collected from the axillary region of untreated mice often presented with small aggregates of leukocytes as demonstrated through flow cytometric assessment of human CD45, CD3, CD4, CD8, CD14, and CD19 as illustrated in Figure 1 and Supplementary Figure 1.  In mice treated with rFTL-3L, larger and more organized lymph node-type structures could be visualized. Shown are brightfield microscopy images, captured at 10X magnification, of HIS mouse lymphoid tissue visualized with H&E.


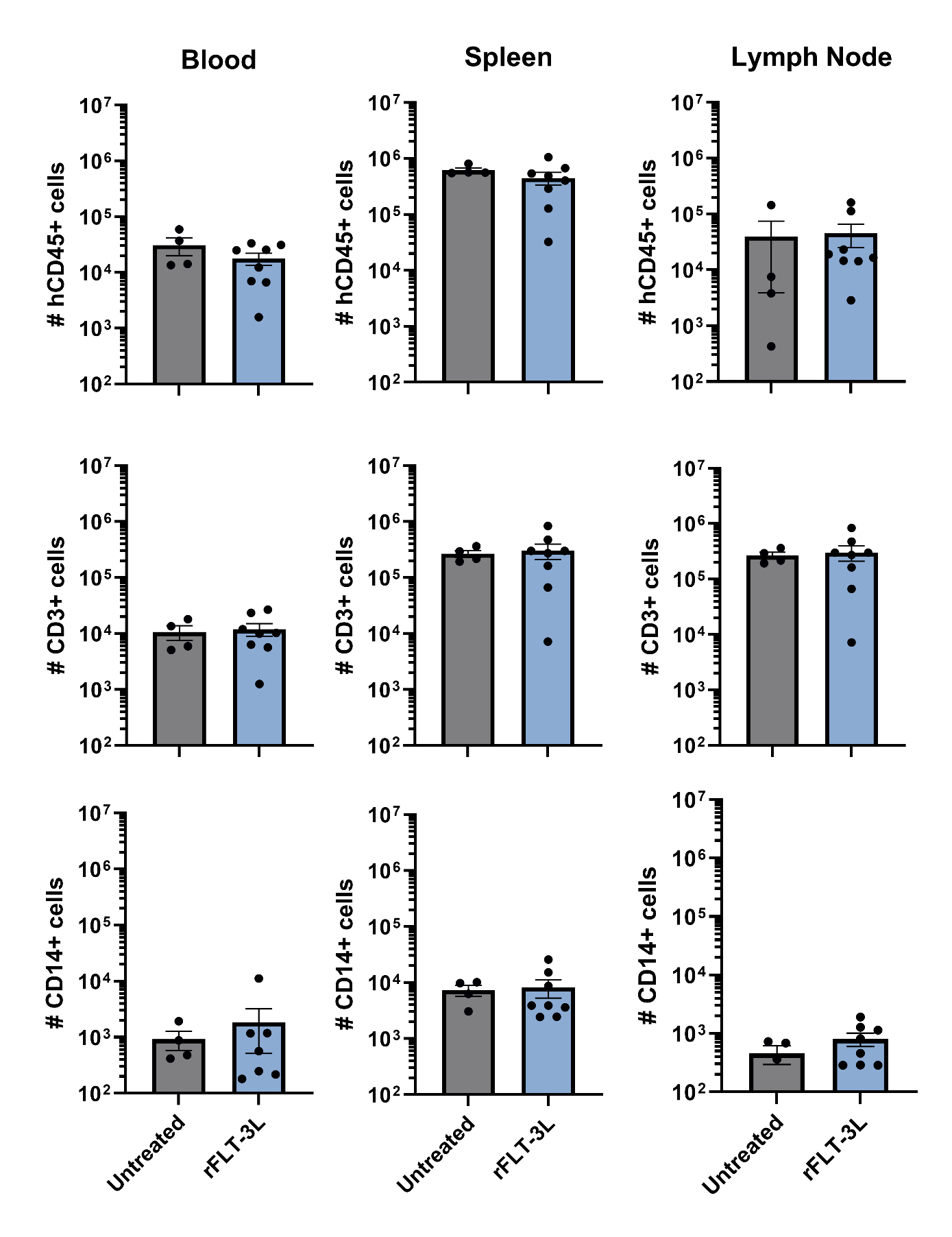


**Figure S2 Recombinant FLT-3L treatment has no effect on human leukocyte numbers in HIS mice.**HIS mice were treated with rFLT-3L as described in Fig, 1.  Blood, spleen and LN were collected on day 14 and reduced to single cell suspensions. Flow cytometric analysis was used to determined changes in the number of human CD45^+^ leukocytes, CD3^+^ T cells and CD14^+^  cells as a result of rFLT-3L treatment. Shown are summarized results following exclusion of doublets and gated selection of cells with side scatter and forward scatter properties of leukocytes and human CD45^+^ phenotype. Values shown are mean ± SEM of individual animals in each group.


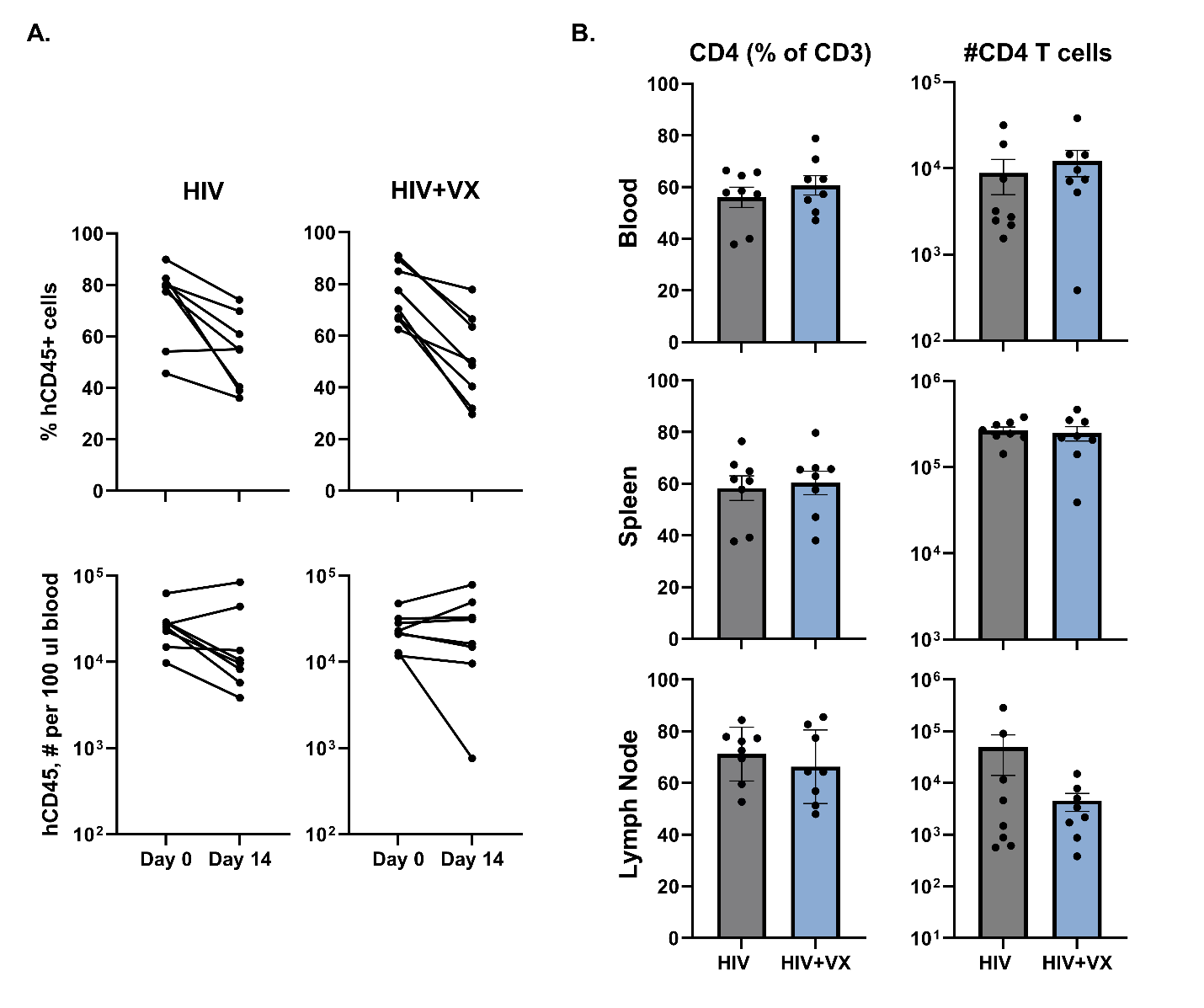


**Figure S3 VX-765 does not alter CD4^+^ T cell populations in HIV-infected mice with moderate leukocyte depletion .** HIS mice were selected for similar human immune system reconstitution and divided into two groups (n=8/group), then infected with HIV-1_ADA_ and treated with VX-765 as described in Fig 3A. Flow cytometric analysis was used to determine (A) changes in the number and percent of CD45^+^ leukocytes in peripheral blood before (d 0) and after (d 14) HIV infection and VX-765 treatment, as well as (B) CD4^+^ T cells in blood, spleen and LN after treatment with VX-765 or no treatment. In the absence of significant HIV-induced leukocyte depletion, there was no effect of VX-765 treatment.


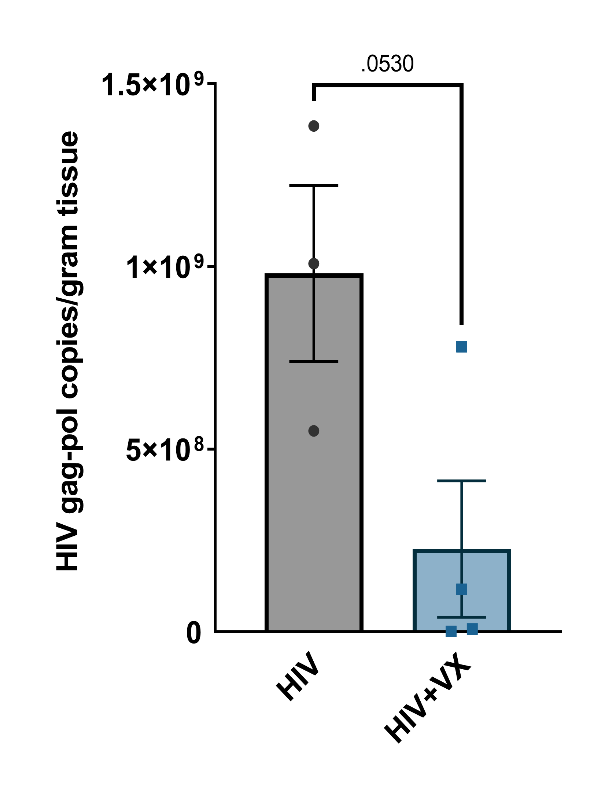


**Figure S4 VX-765 treatment effect on HIV replication in the spleen.**

RNA was extracted from spleen of HIV-infected HIS mice that were either treated with VX-765 or left untreated. Expression of HIV transcripts was assayed by qRT-PCR of HIV *gag*. Level of expression was determined by comparison to known plasmid standards, and normalized to harvested tissue weight. Data shown is the mean ± SEM of individual animals. Differences due to treatment were determined using a two-tailed Student’s T test.
